# Dual antiplatelet therapy increases intracerebral hemorrhage and edema after controlled cortical impact and can be partially encountered by 12/15-lipoxygenase inhibition

**DOI:** 10.1177/0271678X251371376

**Published:** 2025-09-15

**Authors:** Franziska Lieschke, Yi Zheng, Josephine Lok, Jan Hendrik Schaefer, Christian Foerch, Klaus van Leyen

**Affiliations:** 1 Department of Neurology, Goethe University Frankfurt, University Hospital, Frankfurt am Main, Germany; 2 Neuroprotection Research Laboratories, Massachusetts General Hospital, Harvard Medical School, Boston, MA, USA; 3 Department of Pediatrics, Massachusetts General Hospital, Harvard Medical School, Boston, MA, USA; 4 Department of Neurology, RKH Klinikum Ludwigsburg, Ludwigsburg, Germany

**Keywords:** Antiplatelet therapy, aspirin, clopidogrel, lipoxygenase, traumatic brain injury

## Abstract

Traumatic brain injury (TBI) is increasingly prevalent in older age groups, many of whom receive dual antiplatelet therapy (DAPT). The impact of DAPT on post-traumatic intracranial hemorrhage (ICH) and mortality remains controversial. This study investigates ICH in a mouse TBI model under DAPT and explores the potential protective effects of 12/15-lipoxygenase (LOX) inhibition. Male C57BL6 mice received aspirin and clopidogrel in drinking water for 3 days before TBI induction via controlled cortical impact (CCI). The 12/15-LOX inhibitor BPN-27332 was administered i.p. 1 h after CCI. ICH and edema volumes were quantified 24 h post-injury, functional outcomes were assessed over 7 days, and lesion volumes were analyzed on day 7. DAPT significantly increased ICH (36.56 ± 7.1 vs 6.72 ± 2.12 mm^2^, *p* = 0.0004) and edema (21.86% ± 2.0% vs 5.94% ± 2.04%, *p* = 0.0002). BPN-27332 reduced ICH (29.03 ± 4.97 vs 43.99 ± 4.54 mm^2^, *p* = 0.038) and edema (7.98% ± 4.61% vs 26.11% ± 3.76%, *p* = 0.0064) in mice under DAPT. No significant functional differences were observed. Lesion volumes tended to be smaller in the BPN-27332 treated mice. DAPT exacerbates ICH risk in experimental TBI, while 12/15-LOX inhibition may help reduce post-traumatic ICH and edema.

## Introduction

Traumatic brain injury (TBI) represents a leading cause of death and disability worldwide. Particularly young people are affected, but also increasingly, the elderly,^1–3^ a relevant percentage of whom take antiplatelet therapy. This is particularly important, as elderly patients are more likely to be hospitalized and die from TBI compared with all other age groups.^4^ In parallel with the increased age of the individuals who experience TBI, falls became the leading cause for TBI.^5^ Since the primary brain injury takes effect instantly, the only therapeutic option is to address progressive secondary hemorrhage and secondary ischemic injury, which occurs in 30% to over 70% of patients.^6–10^ Measures such as optimizing coagulation (e.g. reversal therapies in anticoagulation-associated hemorrhages), consistent blood pressure management and, if necessary, surgical approaches are currently applied for this purpose. This is important, as hemorrhage progression of contusion predicts unfavorable clinical outcomes.^11–13^

Given the increasing age of TBI patients, comorbidities and the number of patients treated with antiplatelet agents rise equally. Thus, the elderly are more susceptible to bleeding in general and may have an increased risk for intracerebral hemorrhage (ICH) following TBI.^14^ As studies on preinjury antiplatelet intake of aspirin (ASA) putting the patients at higher risk for ICH and delayed bleeding remain controversial,^15–18^ the data on the antiplatelet agent clopidogrel (CPG) suggest preinjury intake as an independent predictor of (progressive) ICH, delayed bleeding, and worse outcomes.^19–21^ Accordingly, a recent meta-analysis identified patients on dual antiplatelet therapy (DAPT) at high risk for delayed bleeding even after mild TBI,^22^ which is in line with other reports linking DAPT to an increased risk of hemorrhage after trauma to the head.^23,24^

Various lipoxygenases have been shown to affect the pathophysiology of brain injury. As we and others have previously shown, 12/15-lipoxygenase (12/15-LOX) plays a significant role in secondary brain damage after various types of acute ischemic and hemorrhagic conditions.^25–29^ The suspected underlying mechanism how lipoxygenases aggravate brain injury include increased leakage of the blood–brain barrier and pronounced neuronal cell death. Furthermore, Kenny et al. suggested that the increased 15-LOX-2 expression in the pericontusional cortex following CCI^30^ contributes to ferroptotic cell death in mice. While 12/15-LOX inhibition has been proven to be protective in various models of ischemic and hemorrhagic stroke,^25–28,31^ 5-LOX inhibition demonstrated beneficial effects in experimental TBI.^29,32,33^ To date, no studies have investigated the interaction of antiplatelet pre-medication and a possible beneficial effect of 12/15-LOX inhibition.

The aim of the present study was therefore to characterize ICH occurring after TBI in a mouse model of DAPT with ASA + CPG. In addition, a protective role of 12/15-LOX inhibition was to be determined.

## Methods

### Mice and pretreatment

Male C57BL6/J mice (Jackson Laboratory) aged 8 weeks with a mean body weight of 26 ± 2 g were used. Aspirin (Bayer Health Care) and clopidogrel (McKesson) were given orally after an established protocol.^34,35^ Briefly, tablets were crushed and dissolved in 300 mL drinking water (concentration: ASA 0.4 mg/mL, CPG 0.15 mg/mL), which was provided to the mice ad libitum for a treatment period of 72 h. The solution was refreshed every second day. Based on an assumed water consumption of 15 mL/100 g/24 h, the estimated daily intake per mouse was 60 mg/kg ASA and 22.5 mg/kg clopidogrel.

### Study design

The first part of our study was designed to compare hemorrhage volume, edema, and neurologic outcomes 24 h after controlled cortical impact (CCI) between ASA + CPG-pretreated and control mice. Control mice received regular drinking water. The second part investigated the effect of 12/15-LOX inhibition by the compound BPN-27332. All mice were pretreated with ASA + CPG before CCI. Mice were then treated with BPN-27332 (120 µL of a solution containing 10 mg/mL, yielding a dosage of 40 mg/kg, administered intraperitoneally (i.p.) 1 h after the impact) and compared to vehicle treated mice (120 µL of dimethyl sulfoxide (DMSO), administered likewise i.p. 1 h after the impact). Here again, hemorrhage volumes, edema, and neurologic outcome were determined 24 h after CCI. The third part included a replication of the previous (second) experiment with the addition of more comprehensive functional tests and the determination of the final lesion volume on day 7 after CCI. The 40 mg/kg dose of BPN-27332 was selected based on internal findings from an experimental stroke model, in which this dose conferred superior neuroprotection compared to 20 mg/kg, without inducing observable side effects. The operators performing surgical procedures (CCI and injections) and behavior tests, and the investigators evaluating data were blinded to the treatment groups.

All animal experiments were conducted in accordance with a protocol approved by the Massachusetts General Hospital Institutional Animal Care and Use Committee. The study adhered to the ethical principles outlined in the Guide for the Care and Use of Laboratory Animals, Eighth Edition, 2011, published by the National Research Council of the National Academies. Furthermore, the reporting of animal experiments complied with the ARRIVE (Animal Research: Reporting of In Vivo Experiments) guidelines 2.0 to ensure transparency and reproducibility in animal research.

### Controlled cortical impact (CCI)

Mice were anesthetized with isoflurane and positioned in a stereotactic frame. A 5-mm craniotomy was made using a portable drill and a trephine over the left parietotemporal cortex, and the bone flap was removed. CCI was performed using a pneumatic cylinder with a 3-mm flat-tip impounder, 5 m/s velocity, 0.5 or 1.5 mm depth, and 150 ms impact duration. Subsequently, the bone flap was not replaced in order to limit the confounding effects of elevated intracranial pressure.^36^ All mice were then allowed to recover in a pre-heated incubator (at 29 °C) and were conscious and able to move after ~5 min.

### Behavior tests

In the first part of the study, functional deficits were assessed 24 h after CCI by using the modified Neurologic Severity Score (NSS), a standard foot fault test and additionally, an open field test (OF). In the second part of the study, behavior tests were performed pre-injury at baseline (in the morning on the day of surgery) and again assessed 24 h after CCI, this time by using the Garcia Score, NSS, the foot fault test and additionally, a standard hanging wire (“wire grip”) test. Lastly, in the third part of the study, functional testing was performed at baseline (NSS, wire grip, foot fault, Garcia Score, and OF), on day 1, 3, and 5 after CCI (NSS, wire grip, foot fault, and Garcia Score), and at the end of the experiment on day 7 after CCI employing the NSS, wire grip, foot fault, Garcia Score, the novel object recognition task (NORT), and OF as explained below.

### Neurological Severity Score (NSS)

The NSS measures neurologic deficits on a 10-point ordinal scale,^37^ consisting of different motor, behavioral, and spontaneous locomotion tasks (for details see Supplemental Data).

### Foot fault

Mice were placed on an elevated grid floor (30 × 20 cm) and videotaped from the side as they traverse the grid. The openings in the grid were 1 × 1 cm. The number of “foot faults” for each forelimb and the time until 100 forelimb steps were recorded. A foot fault index (forelimb faults/100 steps) was calculated.

### Open field (OF)

An open field Plexiglas chamber was used, in which four lines were drawn to delineate nine fields. Mice were placed in the chamber for a minimum of 3 min to acclimate. Then a video was recorded for 5 min, assessing the time spend in the center field versus the outer edge. In addition, the number of fields explored by the mice for 5 min was manually counted.

### Wire grip score

The mice were placed on a metal wire (45 cm long) suspended 45 cm above a foam pad and were allowed to traverse the wire for 60 s. The latency that a mouse remained on the wire within this 60 s interval was measured, and wire grip scores were quantitated using a five-point scale.^36^ The wire grip test was performed in triplicates and an average value per parameter was calculated for each mouse on each day of testing.

### Garcia Neuroscore

The modified Garcia Neuroscore measures neurologic deficits on an 18-point ordinal scale and is composed of six subtests (spontaneous activity, limb extension, forepaw outstretching, climbing, side stroking, and vibrissae touch (for details see Supplemental Data).^38,39^

### Novel object recognition task (NORT)

The NORT was carried out as previously described.^40^ Briefly, after a first habituation episode of 10 min in an empty cage, a single mouse was presented to two identical objects to familiarize with them for the duration of 5 min. Afterwards, the mouse was returned to its home cage for 30 min. Subsequently one familiar object was replaced with a novel object for testing the recognition memory. This retention period was videotaped for 5 min. The time with the new and the familiar object was recorded and a recognition performance (time new object/total time spend on both objects − 0.5) was calculated. The box and the objects were cleaned properly after every mouse. The objects used were differently shaped plastic toys. Sitting on the object without movement was not included in the exploratory activity (time with objects).

### Hemorrhage assessment

After behavior assessment, deeply anesthetized mice were euthanized by transcardial perfusion with cold saline (10 mL). The brains were extracted, sectioned into 1 mm thick coronal sections with a brain matrix and photographed. Using NIH ImageJ software, hemorrhage areas were outlined and measured as red areas in brain sections. The total intracerebral hemorrhage area was determined as the sum of hemorrhagic areas in all coronal brain sections. The brain sections were then separated into ipsi- and contralateral hemispheres placed in microcentrifuge tubes containing 0.1 mL saline. A tissue grinder was used for homogenization and 0.9 mL saline were added. The samples were vortexed, and ultrasound was applied for 1–5 s to lyse erythrocytic cell membranes. After centrifugation for 30 min (13,000*g* at 4 °C), triplicates of 100 µL of supernatant were added to 40 µL of Drabkin’s reagent in a 96 well plate. After incubation for 15 min at room temperature, absorption rates were determined at 540 nm using a standard plate reader. Hemorrhage volumes in microliters (µL) were calculated from mean absorbance rates using a standard curve generated by titrating perfused control brain homogenates with defined volumes of whole blood from uninjured mice (0.5–20 µL), resulting in the equation: *Y* = 0.03291 × *X* + 0.08359.

### Assessment of brain edema

The extent of cerebral edema was measured in the same pictures taken for the hemorrhage area measurements, again using NIH ImageJ software. The volumes of both hemispheres were calculated in pixels from the summation of coronal slice areas. Brain edema was expressed as a percentage of the normal areas in the contralateral unaffected hemisphere, calculated using the Kaplan method: extent of edema = (the volume of right hemisphere − the volume of left hemisphere)/the volume of left hemisphere).^41,42^

### Assessment of lesion volume and brain tissue loss

At the end of the study (after behavior assessment on day 7), deeply anesthetized mice were euthanized by transcardial perfusion with PBS and 4% PFA. Subsequently, brains were extracted and photographed. The brains were then immersed in 4% PFA overnight at 4 °C, and cryoprotected in 15% and 30% sucrose solutions in PBS at 4 °C before freezing. Frozen coronal sections (20 μm thick) were prepared using a cryostat at 0.5-mm intervals from the anterior to the posterior and mounted on Fisherbrand™ Superfrost™ Plus Microscope Slides. Sections were stained with hematoxylin and eosin according to the manufacturer’s protocol (Vector Laboratories, H-3502). Whole sections were photographed and images were analyzed using NIH ImageJ software. The area of the lesion was quantitated using free hand selection and the lesion volume was expressed in mm^3^. Brain tissue loss was calculated by obtaining the total volume of remaining tissue in the right (uninjured) and left (injured) hemispheres. Percent tissue loss was calculated as volume of the ((Right hemisphere − Left hemisphere)/Right hemisphere) × 100%.

### Statistics

Data was statistically analyzed using GraphPad Prism 10 (version 10.1.0). Metric data was assessed by *t*-test or Mann–Whitney *U* test if the Kolmogorov–Smirnov test was negative for normal distribution. Correlations were performed using Spearman *r* and represented by rho (*r*). In the third part of the study, comparisons between two groups over different time points were conducted using multiple Mann–Whitney tests with application of a false discovery rate using a two-stage step-up method (Benjamini, Krieger, and Yekutieli). Survival distribution was analyzed using Kaplan–Maier curves with log-rank (Mantel–Cox) and Gehan–Breslow–Wilcoxon test. The significance level for all tests was set at *p* < 0.05.

## Results

### ASA + CPG pretreatment increases bleeding and edema volumes after CCI

**Figure 1.**
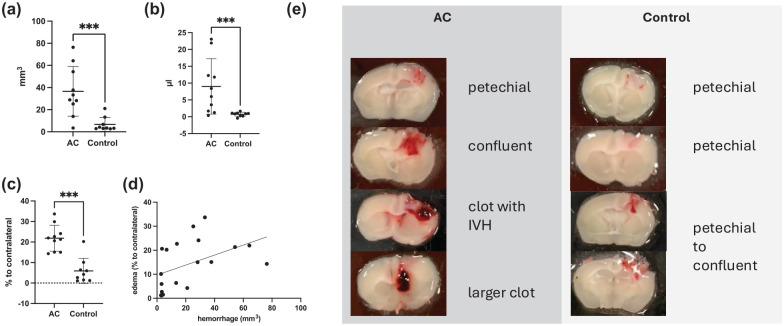
(a) Hemorrhage measured using ImageJ in brain sections 24 h after controlled cortical impact (CCI) and (b) using a standard hemoglobin assay. (c) Perilesional edema. (d) Correlation of edema formation with hemorrhage expansion. (e) exemplary macroscopic images of the hemorrhages observed. AC: aspirin and clopidogrel pretreated mice; control: mice were not treated prior to CCI; IVH: intraventricular hemorrhage; *p < 0.05, **p < 0.01, and *** *p* < 0.001.

In the first experiment, in total 20 mice (11 ASA + CPG-pretreated and 9 untreated control mice) were subjected to mild CCI (0.5 mm depth). Functional outcome, hemorrhage and edema volume was assessed 24 h after CCI. Mortality was 5% (one ASA + CPG-pretreated mouse deceased), leaving 9 control and 10 ASA + CPG pretreated mice to be included in the evaluation (for details please see Supplemental Table 1). As expected, untreated control mice showed very little hemorrhage (measured visually 6.72 ± 2.12 mm^2^, and by hemoglobin-assay 0.72 ± 0.21 µL, respectively). Mice receiving ASA + CPG pretreatment showed significantly increased hemorrhage compared with control mice (36.56 ± 7.1 mm^2^, *p* = 0.0004; 9.02 ± 2.61 µL, *p* = 0.0079, respectively, Figure 1(a) and (b)) with a considerable variability in the morphology of the hemorrhages. Specifically, some mice exhibited minimal bleeding (petechial) confined to the impact site, while others developed more extensive, confluent hemorrhages, including bleeding at sites distant from the primary impact (contrecoup, exemplary images in Figure 1(e)). Ipsilateral edema was increased by 15.93% ± 2.87% in ASA + CPG pretreated mice as compared to untreated control mice (Figure 1(c), *p* = 0.0002). Development of hemorrhage was associated with the occurrence of edema in these experiments (spearman r = 0.64, *p* = 0.0035, *n* = 19, Figure 1(d)). Body weight and neurological outcome at 24 h after CCI, measured by open field (number of fields/5 min), foot fault (time until 100 steps, number of right forelimb errors), and NSS, was not different between groups (data shown in the Supplemental Data).

### 12/15-LOX inhibition by BPN-27332 reduces bleeding and edema volumes after CCI in ASA + CPG pretreated mice

**Figure 2.**
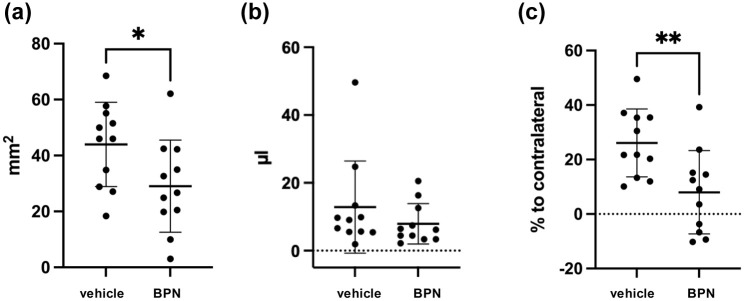
(a) Hemorrhage measured manually in brain sections 24 h after controlled cortical impact (CCI) and (b) using a standard hemoglobin assay. (c) Perilesional edema. BPN: mice treated with the 12/15-LOX inhibitor BPN-27332; *p < 0.05, **p < 0.01, and ***p < 0.001.

To reduce the variability in bleeding outcomes observed in the previous experiment, we increased the impact depth, which resulted in more consistent hematoma formation without compromising surgical feasibility. In total 28 mice (all ASA + CPG-pretreated) were subjected to moderate CCI (1.5 mm depth). One hour after the impact, BPN-27332 or its vehicle DMSO were given i.p. (*n* = 14/treatment group). Functional outcome, hemorrhage, and edema volume were assessed 24 h after CCI. Due to the higher level of impact in this more severe version of the model, mortality was 18% (three vehicle and two BPN-27332 treated mice deceased), in addition one BPN-27332 treated mouse had to be excluded due to a surgical error, leaving 11 mice/group to be included in the evaluation (for details please see Supplemental Table 1). BPN-27332 treated mice tended to have less bleeding than vehicle treated mice (measured via ImageJ −14.95 ± 6.73 mm^2^, *p* = 0.038, Figure 2(a) and by hemoglobin-assay −4.93 ± 4.47 µL, *p* = 0.3, Figure 2(b)). However, edema was significantly reduced by 18.12% ± 5.95% in BPN-27332 treated mice as compared to vehicle treated mice (*p* = 0.0064, Figure 2(c)). Again, body weight and neurological outcome at 24 h after CCI, measured by wire grip test (latency and score), Garcia score, foot fault (time until 100 steps, number of right forelimb errors), and NSS, were not different between groups (data shown in the Supplemental Data).

### BPN-27332 has a neutral effect on functional outcome in the subacute phase but tends to reduce lesion size in ASA + CPG pretreated mice at day 7 following CCI

**Figure 3.**
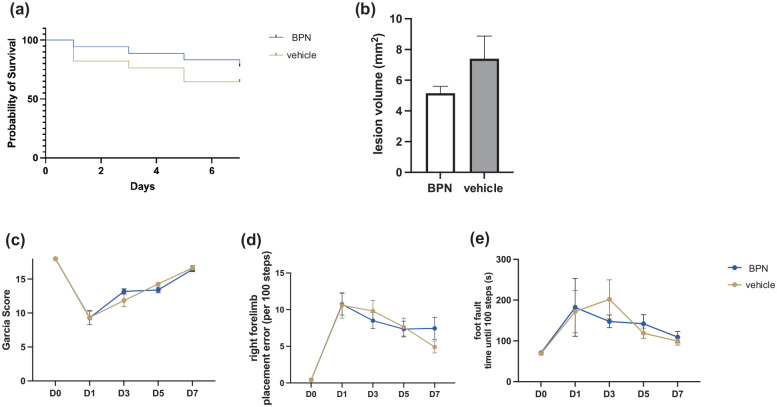
(a) Kaplan–Meier curves of survival and (b) lesion volumes measured at day 7 after the controlled cortical impact. Behavior testing was performed at baseline, as well as at day 1, 3, 5, and 7 after the experimental traumatic brain injury. Represented in excerpts, Garcia Neuroscore (c), mean right forelimb error rate (d), mean latency until 100 steps reached (e). Neuroscores were graphically represented with standard error of the mean. BPN: BPN-27332 treated mice.

In the third experiment, in total 36 mice (all ASA + CPG-pretreated) were subjected to moderate CCI (1.5 mm depth). One hour after the impact, BPN-27332 or its vehicle DMSO were given i.p. Functional outcome was assessed at baseline as well as at day 1, 3, 5, and 7 after CCI. Overall mortality was 27.78% (six vehicle and four BPN-27332 treated mice deceased by day 7); in addition one vehicle-treated mouse had to be excluded due to an impactor malfunction. In the end, 14 BPN-27332 treated mice and 11 vehicle treated mice were included in the evaluation (for details please see Supplemental Table 1). Although mortality was higher in the vehicle-treated group, the Kaplan–Meier curves did not differ significantly (log-rank test χ^2^ = 0.8270, *p* = 0.3631, Figure 3(a)). No functional differences could be observed in the subacute phase, but there was a trend of—2.25 ± 1.43 mm^2^ towards smaller lesion volumes in the BPN-27332 treated mice, however this difference was not statistically significant (mean lesion size BPN-27332 vs vehicle: 5.15 ± 0.45 vs 7.4 ± 1.47, *p* = 0.1674, Figure 3(b)). All mice showed a significant increase in the NSS on the first day after CCI when compared to baseline, which gradually returned almost to the initial value on day 7 after CCI (Supplemental Data). A similar result was seen for the Garcia score—a poorer performance on the first day and a gradual improvement to almost baseline on day 7 (Figure 3(c)). In the wire grip test, all mice exhibited a significant reduction in the latency to hold on to the wire on day 1 after CCI, which increased again in the following days and almost reached the baseline on day 7. Similarly, the mean wire grip scores improved again over the course of the observation period after an initial drop (Supplemental Data). In the foot fault test, when walking over a grid, all mice showed a significant increase in the rate of right forelimb slips on the first day after CCI; in addition, the time required for a mouse to cover 100 steps on the grid was significantly increased (Figure 3(d) and (e)). However, the mice recovered equally over the observation period, but without returning to their initial baseline again. Following CCI, mice expressed a reduced number of fields explored in the open field test, which was slightly emphasized in BPN-27332 treated mice compared to vehicle treated mice and went along with a slightly more reduced center time ratio, maybe indicating a more anxious phenotype. In the novel object recognition task, there was no difference in the exploratory behavior or memory between the two groups (Supplemental Data).

## Discussion

In summary, we found that ASA + CPG significantly increased ICH following experimental TBI, which went along with increased ipsilateral swelling and thus a higher risk for mortality and clinical deterioration. To the best of our knowledge, there is no published preclinical data on TBI-related ICH during DAPT in mice. However, this is of importance because modeling clinical scenarios in rodents allows for a controlled environment and standardized conditions, so that mechanistic studies and more importantly, testing platforms for possible strategies reducing hemorrhagic complications can be established. A previous study by our group demonstrated the feasibility of such investigations. In a mouse model of TBI employing CCI during different regimes of oral anticoagulation, we were able to demonstrate that pretreatment and anticoagulation with warfarin but not with dabigatran caused increased ICH 24 h after TBI.^43^ In line with the clinical observations, linking DAPT to a high risk for delayed bleeding after TBI,^22–24^ our study confirmed this in a translational model for the very first time. More importantly, we found 12/15-LOX inhibition to partially reverse these findings, introducing its application as an additive treatment strategy in TBI. TBI can be produced by heterogeneous impacts causing a primary injury, which is then followed by complex secondary responses.^44–46^ Whereas the primary impact cannot be addressed other than in prevention in the first place, therapeutic approaches focus on the secondary injury. Among other mechanisms, oxidative stress is a major driver in secondary damage causing a dysregulation of the blood-brain barrier (BBB). Thus, stabilizing the BBB using modulators of inflammation and antioxidants represents an important strategy. Inhibiting 12/15-LOX was previously shown to reduce oxidative stress, BBB breakdown and thus edema reduction in various models of brain damage such as ischemic stroke and ICH.^27,28,31,47,48^ Our present work further confirmed these observations. However, we stated that 12/15-LOX inhibition only partially reversed the effects of increased ICH and edema following CCI under DAPT, as our observations are mainly based on the visual assessment using the Image J software. We therefore supplemented the evaluation with the photometric hemoglobin assay method, which is assumed to be more objective; although the results remained not significant in the second part of our study. This is probably due to the lower sensitivity of this method for small volumes, as the processing of the sample leads to waxing effects and losses, making it difficult to detect differences between the groups at very low volumes. Nevertheless, the results on day 7, with the lesions tending to be smaller, suggest that the observed effects may be genuine. The fact that the difference here was not statistically significantly different is likely due to the small sample size, which is explained by the increased mortality rate of the model at the selected severity (“moderate CCI” with 1.5 mm impact penetration depth). While out of the scope of the current study, our findings may have important implications to anticoagulated mice too, given that TBI induces complex systemic effects on the coagulation system.^14,49,50^ In the context of TBI combined with anticoagulation, specifically with vitamin k antagonists like warfarin, the risk of ICH is increased^51^ and delayed bleeding might occur more frequently.^52^ Since LOX-inhibition, especially when initiated after traumatic impact, does not affect the primary injury (i.e. initial bleeding), and is unlikely to directly influence hemostasis, any beneficial effects are most likely mediated through stabilization of the BBB^25,31^ and antiinflammatory properties via an antioxidative mechanisms.^53–55^ Supporting this, 12/15-LOX deletion or inhibition significantly reduced hemorrhagic transformation and edema in a mouse model of middle cerebral artery occlusion under warfarin, independent of infarct size.^27^

There are several limitations to our work. Our studies were conducted in the acute and subacute phase only. We focused on this early phase because it is clinically the most decisive, in which neurological deterioration and complications such as hemorrhage progression, epileptic seizures and intracranial pressure have a significant impact on survival.^8^ The chosen behavior tests were neutral between the different treatment groups on the first day, and over the course of the initial 7 days following CCI, which applied to the model itself and the therapeutical studies. It is perplexing that clinical improvement in edema volume was seen in the BPN treated mice at 24 h after CCI, yet a worse functional outcome was seen in this group in the Open Field test at 7 days after CCI. This discrepancy may be due to limitations of the small sample size of this study. In the clinical context, there is no question that increased bleeding and edema are associated with more complications, poorer survival and an increased mortality rate.^11–13,56^ The finding that the BPN-27332-treated mice spent slightly less time in the center of the field than the vehicle-treated mice (*p* = 0.0665) and exhibited less exploratory behavior overall post-injury may indicate that the treated mice had more anxiety, which may be a general adverse effect of the medication, unrelated to its effect on the pathophysiology after TBI. This indicates that the treated mice appear to be somewhat more anxious. Whether this is a true observation and how it can be explained must be investigated in further studies. Furthermore, this study lacks mechanistic studies, explaining the underlying factors and supporting our hypothesis, that 12/15-LOX inhibition stabilizes the BBB and thus prevents progression of bleeding and edema formation. As edema and hemorrhage post-TBI represent partially independent processes, with further mechanistic studies missing, we were unable to fully delineate which underlying pathway contributed predominantly to our observed effects. However, based on prior data from our group, we know that platelet activation assays in LOX-knockout and wild-type mice under ASA + CPG treatment showed similarly reduced platelet activation, indicating preserved DAPT efficacy. Moreover, LOX-inhibition did not affect warfarin-induced anticoagulation (as measured by INR) or bleeding outcomes in a standard tail bleeding assay under DAPT following MCAO. These findings suggest that LOX-inhibition does not compromise anticoagulant or antiplatelet effects. Given the complex interplay between platelets and leukocytes in post-traumatic inflammation,^57,58^ and previous evidence that 12/15-LOX contributes to secondary injury via macrophage activation,^28^ we propose that combining DAPT with LOX-inhibition may synergistically attenuate inflammation and improve outcomes after TBI.^59,60^ Further studies are therefore urgently needed. Lastly, our study was conducted in young males only (in order to minimize confounding effects linked to hormonal fluctuations). There is a lack of data on female and older mice as well as its application in a disease model with comorbidities such as arterial hypertension or diabetes. However, the chosen simplification represents a reliable and standardized approach to which relevant factors such as age, sex, comorbidities, and mechanism of injury (blunt vs penetrating) could be added for methodical evaluation of each factor.

## Conclusions

DAPT contributes significantly to the increased risk of post-traumatic ICH and edema formation. Therapeutically, 12/15-LOX inhibition may represent an additional strategy to treat such complications. However, future studies are needed to further clarify the role of lipoxygenase in this context, especially with regard to improving clinical outcomes.

## Supplemental Material

sj-pdf-1-jcb-10.1177_0271678X251371376 – Supplemental material for Dual antiplatelet therapy increases intracerebral hemorrhage and edema after controlled cortical impact and can be partially encountered by 12/15-lipoxygenase inhibition Supplemental material, sj-pdf-1-jcb-10.1177_0271678X251371376 for Dual antiplatelet therapy increases intracerebral hemorrhage and edema after controlled cortical impact and can be partially encountered by 12/15-lipoxygenase inhibition by Franziska Lieschke, Yi Zheng, Josephine Lok, Jan Hendrik Schaefer, Christian Foerch and Klaus van Leyen in Journal of Cerebral Blood Flow & Metabolism
