# Supplementary material for: Dual antiplatelet therapy increases intracerebral hemorrhage and edema after controlled cortical impact and can be partially encountered by 12/15-lipoxygenase inhibition
Source: J Cereb Blood Flow Metab. 2025 Sep 15:0271678X251371376. Online ahead of print. doi: 10.1177/0271678X251371376 (PMC12436321; doi:10.1177/0271678X251371376)
Supplement: sj-pdf-1-jcb-10.1177_0271678X251371376 – Supplemental material for Dual antiplatelet therapy increases intracerebral hemorrhage and edema after controlled cortical impact and can be partially encountered by 12/15-lipoxygenase inhibition [file sj-pdf-1-jcb-10.1177_0271678X251371376.pdf]

Supplemental Data

Study part 1: TBI in AC-pretreated vs. untreated control mice

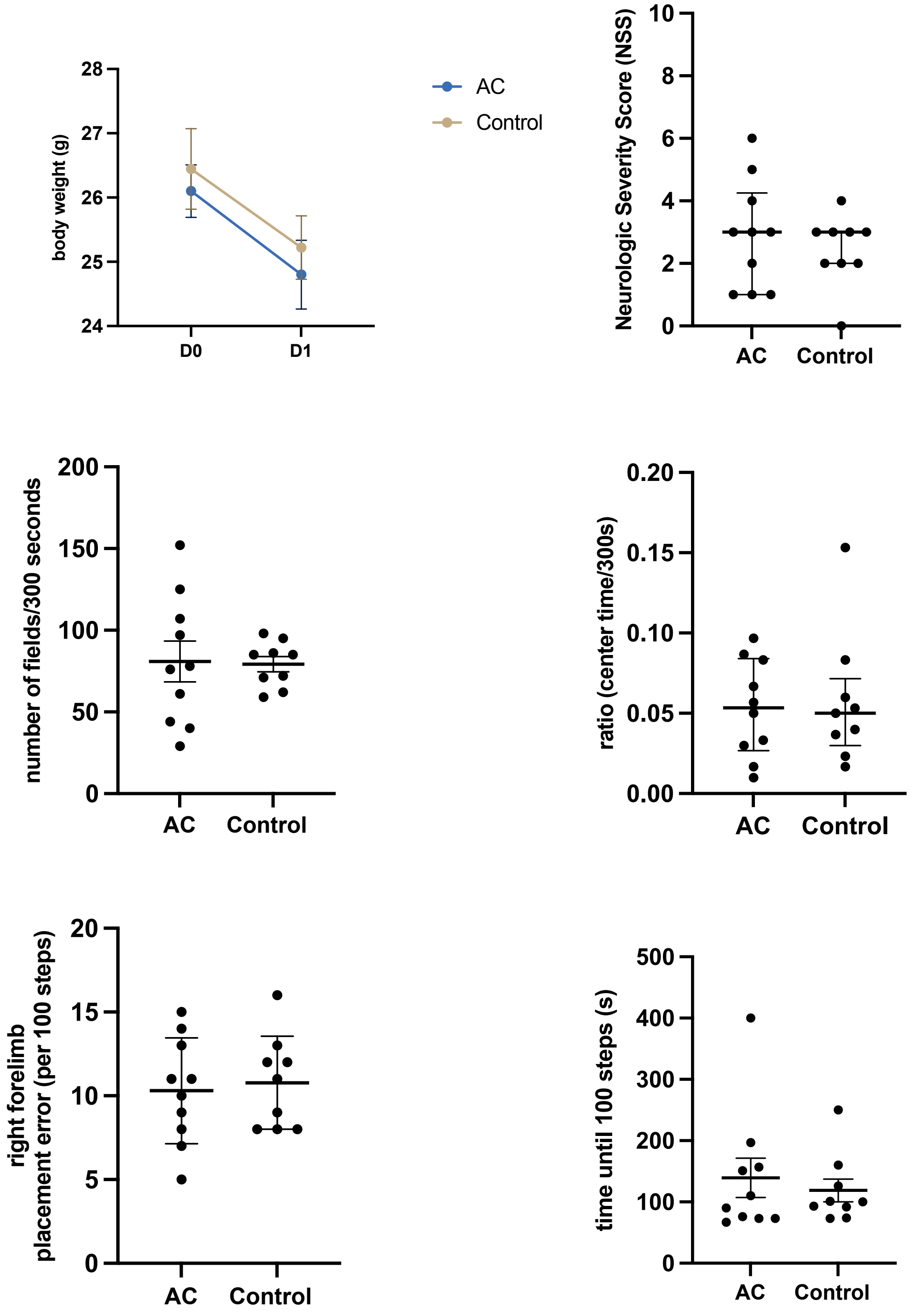

Fig. S1. Body weight and neurological outcome at 24 hours after CCI in AC vs. control mice, measured by open field (number of fields/5minutes), foot fault (time until 100 steps, number of right forelimb errors), and NSS. D0 indicates before TBI; D1 indicates 24h post TBI. Abbreviations: AC=Aspirin and Clopidogrel, CCI= controlled cortical impact, NSS= Neurologic Severity Score, TBI= traumatic brain injury.

Treatment group (number of mice)	Mean ± SEM or median (IQR)	Difference (mean ± SEM)	P value
**Body weight loss (g)**			
AC (n=10)	1.3 ± 0.3	0.0778 ± 0.441	0.8621
Control (n=9)	1.2 ± 0.32		
**Neurological severity Score**			
AC (n=10)	3 (1-4)		0.7055
Control (n=9)	3 (2-3)		
**Open field**			
Number of fields explored per 300 seconds			
AC (n=10)	80.9 ± 12.52	1.68 ± 13.93	0.9056
Control (n=9)	79.22 ± 4.62		
Time in seconds spent in the center per 300 seconds			
AC (n=10)	0.05 (0.03-0.08)		0.9846
Control (n=9)	0.05 (0.03-0.072)		
**Foot Fault**			
Right forelimb placement errors per 100 steps			
AC (n=10)	10.3 ± 1.0	0.48 ± 1.37	0.732
Control (n=9)	10.78 ± 0.93		
Time until 100 steps			
AC (n=10)	139.4 ± 32.16	20.62 ± 38.31	0.8417
Control (n=9)	118.8 ± 18.68		

Table S1: Body weight and neurological outcome at 24 hours after CCI in AC vs. control mice, detailed results of the testing. Abbreviations: AC=Aspirin and Clopidogrel.

Study part 2: LOX-inhibition by BPN-27332 in ASA+CPG pretreated mice following CCI

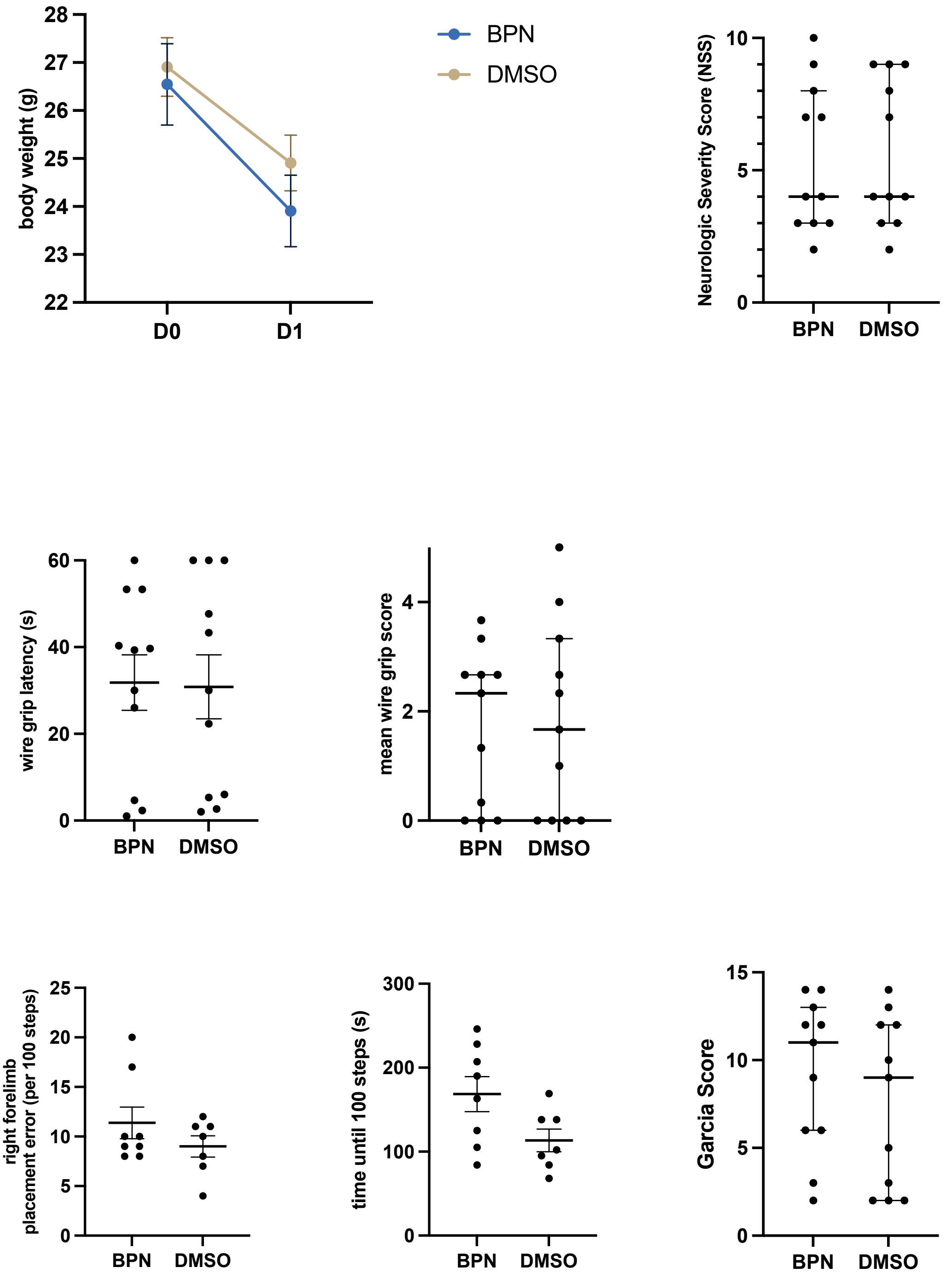

Fig. S2. Body weight and neurological outcome at 24 hours after CCI in ASA+CPG mice treated with the 12/15-LOX inhibitor BPN-27332 vs. vehicle (DMSO), measured by wire grip test (latency and score), Garcia Score, foot fault (time until 100 steps, number of right forelimb errors), and NSS. D0 indicates before TBI; D1 indicates 24h post TBI. Abbreviations: ASA+CPG=Aspirin and Clopidogrel, CCI= controlled cortical impact, NSS= Neurologic Severity Score. BPN= BPN-27332, DMSO= dimethyl sulfoxide, LOX= lipoxygenase.

Treatment group (number of mice)	Mean ± SEM or median (IQR)	Difference (mean ± SEM)	P value
**Body weight loss (g)**			
BPN (n=11)	2.64 ± 0.34	0.64 ± 0.43	0.2769
DMSO (n=11)	2.0 ± 0.27		
**Neurological severity Score**			
BPN (n=11)	4 (3-8)		0.8145
DMSO (n=11)	4 (3-9)		
**Wire grip test**			
Mean wire grip score			
BPN (n=11)	2.33 (0-2.67)		0.9621
DMSO (n=11)	1.67 (0-3.33)		
Mean latency			
BPN (n=11)	31.82 ± 6.39	0.97 ± 9.75	0.8341
DMSO (n=11)	30.85 ± 7.36		
**Foot Fault**			
Right forelimb placement errors per 100 steps			
BPN (n=8)	11.38 ± 1.6	2.38 ± 1.98	0.6688
DMSO (n=7)	9 ± 1.1		
Time until 100 steps			
BPN (n=8)	168.5 ± 20.59	55.07 ± 25.79	0.0769
DMSO (n=7)	113 ± 13.54		
**Garcia Score**			
BPN (n=11)	11 (6-13)		0.3883
DMSO (n=11)	9 (2-12)		

Table S2. Body weight and neurological outcome at 24 hours after CCI in ASA+CPG mice treated with the 12/15-LOX inhibitor BPN-27332 vs. vehcile (DMSO), measured by wire grip test (latency and score), Garcia Score, foot fault (time until 100 steps, number of right forelimb errors), and NSS, detailed results of the testing. Abbreviations: BPN= BPN-27332, DMSO= dimethyl sulfoxide.

Study part 3: Longer term model of LOX-inhibition by BPN-27332 in ASA+CPG pretreated mice following CCI

	D0 (baseline)	D1	D3	D5	D7
Body weight	x	x	x	x	x
NSS	x	x	x	x	x
Wire grip test	x	x	x	x	x
Foot fault test	x	x	x	x	x
Open field	x				x
Garcia Score	x	x	x	x	x
NORT					x

Table S3: overview outcome parameters assessed in the third part of the study. D0 indicates before CCI (baseline); D1-7 indicates days 1-7 post CCI. Abbreviations: ASA+CPG= Aspirin and Clopidogrel, CCI= controlled cortical impact, LOX= lipoxygenase, NSS= neurological severity score, NORT= novel object recognition task.

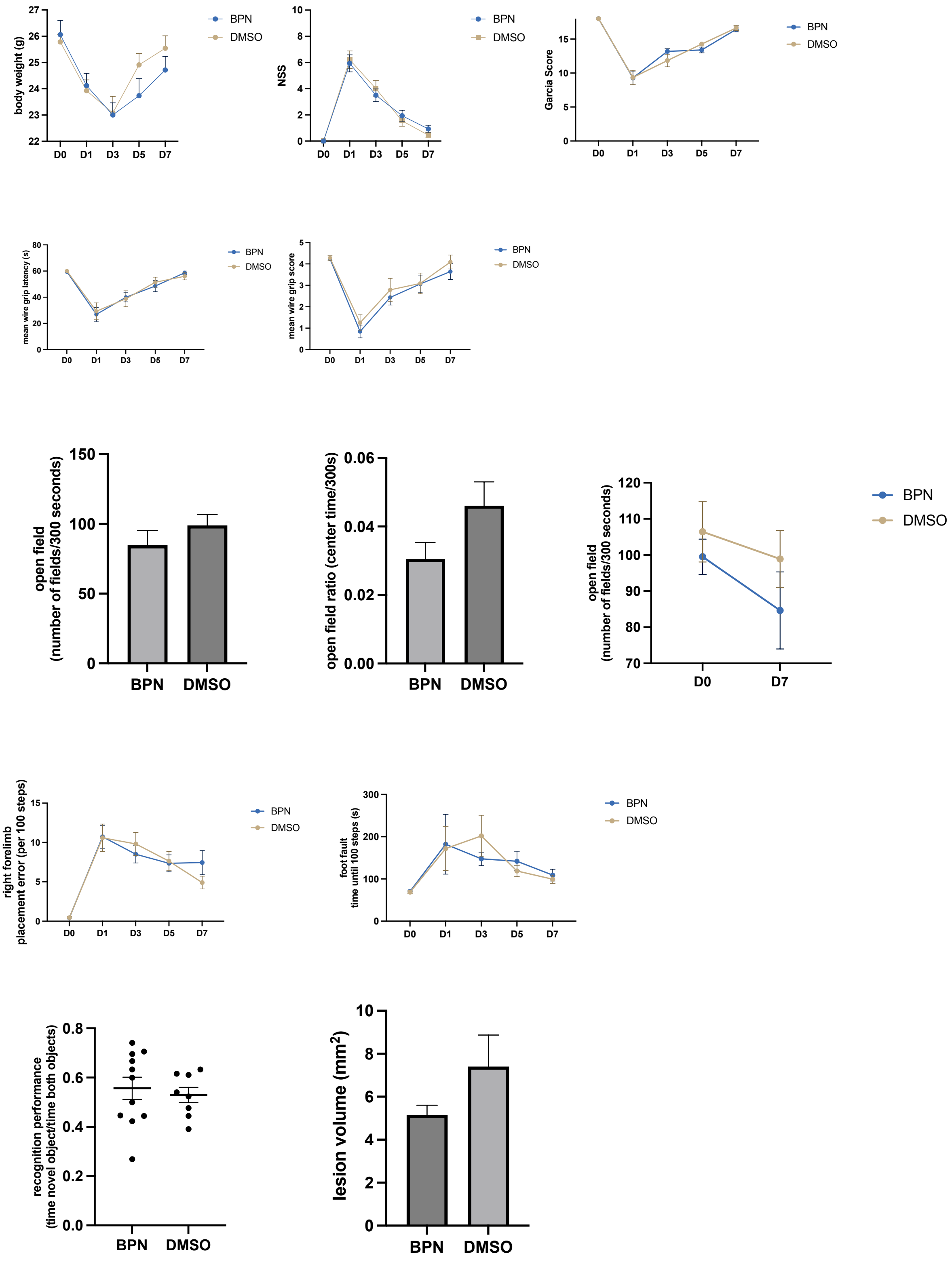

Fig. S3. Body weight and neurological outcome over time and final lesion volume after CCI in AC mice treated with the 12/15-LOX inhibitor BPN-27332 vs. vehicle (DMSO), measured by wire grip test (latency and score), Garcia Score, foot fault (time until 100 steps, number of right forelimb errors), open field, NORT and NSS. D0 indicates before CCI (baseline); D1-7 indicates days 1-7 post CCI. Abbreviations: AC= Aspirin and

Clopidogrel, BPN= BPN-27332, CCI= controlled cortical impact, DMSO= dimethyl sulfoxide, LOX= lipoxygenase, NORT= novel object recognition task, NSS= Neurologic Severity Score.

Treatment group (number of mice)	P value D1	P value D3	P value D5	P value D7
**Body weight (g)**				
BPN	0.914428	0.800657	0.312996	0.413142
DMSO				
**Neurological severity Score**				
BPN	0.885289	0.561106	0.606974	0.179183
DMSO				
**Wire grip test**				
Mean wire grip score				
BPN	0.351477	0.502368	0.826773	0.497108
DMSO				
Mean latency				
BPN	0.659021	0.904536	0.657207	0.341594
DMSO				
**Foot Fault**				
Right forelimb placement errors per 100 steps				
BPN	0.873110	0.579421	0.946687	0.248742
DMSO				
Time until 100 steps				
BPN	0.544727	0.861305	0.657004	0.807649
DMSO				
**Garcia Score**				
BPN	0.882131	0.319043	0.146119	0.853272
DMSO				
**Open field**				
Number of fields explored per 300 seconds				
BPN				0.123050
DMSO				
Time in seconds spent in the center per 300 seconds				
BPN				**0.066496**
DMSO				
**NORT**				
BPN				0.5311
DMSO				

Table S4. Body weight and neurological outcome over time after CCI in AC mice treated with the 12/15-LOX inhibitor BPN-27332 vs. vehicle (DMSO), measured by wire grip test (latency and score), Garcia Score, foot fault (time until 100 steps, number of right

forelimb errors), open field, NORT and NSS. D1-7 indicates days 1-7 post CCI. Abbreviations: AC= Aspirin and Clopidogrel, BPN= BPN-27332, CCI= controlled cortical impact, DMSO= dimethyl sulfoxide, NORT= novel object recognition task, NSS= Neurologic Severity Score.

Study part 1-3

Study Part	Total number of mice	Number of mice per treatment group	deceased	Other exclusions	Included in outcome assessments
I	20	11AC	1 AC		10 AC
		9 Controls			9 Controls
II	28	14 BPN	2 BPN	1 BPN	11 BPN
		14 vehicle	3 vehicle		11 vehicle
III	36	18 PBN	4 BPN		14 BPN
		18 vehicle	6 vehicle	1 vehicle	11 vehicle

Supplemental Table S5. Comprehensive summary of treatment group assignments and mortality in mice. Abbreviations: AC=Aspirin+Clopidogrel, BPN=BPN-27332.
